# MRSA compendium of epidemiology, transmission, pathophysiology, treatment, and prevention within one health framework

**DOI:** 10.3389/fmicb.2022.1067284

**Published:** 2023-01-10

**Authors:** Muhammad Shoaib, Amjad Islam Aqib, Iqra Muzammil, Noreen Majeed, Zeeshan Ahmad Bhutta, Muhammad Fakhar-e-Alam Kulyar, Mahreen Fatima, C-Neen Fatima Zaheer, Afshan Muneer, Maheen Murtaza, Muhammad Kashif, Furqan Shafqat, Wanxia Pu

**Affiliations:** ^1^Key Laboratory of New Animal Drug Project, Gansu Province/Key Laboratory of Veterinary Pharmaceutical Development, Ministry of Agriculture and Rural Affairs/Lanzhou Institute of Husbandry and Pharmaceutical Sciences of the Chinese Academy of Agricultural Sciences, Lanzhou, China; ^2^Department of Medicine, Cholistan University of Veterinary and Animal Sciences, Bahawalpur, Pakistan; ^3^Department of Medicine, University of Veterinary and Animal Sciences, Lahore, Pakistan; ^4^Institute of Microbiology, University of Agriculture, Faisalabad, Pakistan; ^5^Laboratory of Biochemistry and Immunology, College of Veterinary Medicine, Chungbuk National University, Cheongju, Republic of Korea; ^6^College of Veterinary Medicine, Huazhong Agricultural University, Wuhan, China; ^7^Faculty of Biosciences, Cholistan University of Veterinary and Animal Sciences, Bahawalpur, Pakistan; ^8^Faculty of Veterinary Science, University of Agriculture, Faisalabad, Pakistan; ^9^Department of Zoology, Cholistan University of Veterinary and Animal Sciences, Bahawalpur, Pakistan; ^10^Department of Microbiology, Cholistan University of Veterinary and Animal Sciences, Bahawalpur, Pakistan

**Keywords:** MRSA, epidemiology, pathophysiology, transmission, treatment, prevention, MRSA infections

## Abstract

*Staphylococcus aureus* is recognized as commensal as well as opportunistic pathogen of humans and animals. Methicillin resistant strain of *S. aureus* (MRSA) has emerged as a major pathogen in hospitals, community and veterinary settings that compromises the public health and livestock production. MRSA basically emerged from MSSA after acquiring SCC*mec* element through gene transfer containing *mecA gene* responsible for encoding PBP-2α. This protein renders the MRSA resistant to most of the β-lactam antibiotics. Due to the continuous increasing prevalence and transmission of MRSA in hospitals, community and veterinary settings posing a major threat to public health. Furthermore, high pathogenicity of MRSA due to a number of virulence factors produced by *S. aureus* along with antibiotic resistance help to breach the immunity of host and responsible for causing severe infections in humans and animals. The clinical manifestations of MRSA consist of skin and soft tissues infection to bacteremia, septicemia, toxic shock, and scalded skin syndrome. Moreover, due to the increasing resistance of MRSA to number of antibiotics, there is need to approach alternatives ways to overcome economic as well as human losses. This review is going to discuss various aspects of MRSA starting from emergence, transmission, epidemiology, pathophysiology, disease patterns in hosts, novel treatment, and control strategies.

## 1. Introduction

Currently, there are 81 species and numerous subspecies in the genus *Staphylococcus*. The majority of the genus’s species are opportunistic pathogens or commensals of mammals. Numerous species have veterinary as well as medical significance. *Staphylococcus aureus* (*S. aureus*) is among the most prodigious and important staphylococcal species for human pathogenicity (Haag et al., 2019). The name *Staphylococcus* is derived from two Greek words, “staphyle,” which means cluster or bunch, and “kokkos,” means grapes, and so-called as “bunch of grapes” upon observation under microscope. The term “golden staph” is derived from the phrase “*Staphylococcus aureus*” which means “Golden Cluster Seed” (Ogston, 1881). *S. aureus* is a coccus-shaped, gram-positive, non-motile, non-spore-forming, opportunistic bacteria with biochemical profile as catalase, nucleases, lipases, coagulase, catalase, proteases, collagenases, and β-lactamase are the enzymes produced by *S. aureus*. It produces colonies in a variety of colors on various culture media such as pink colonies on chromogenic agar, golden or grayish-white colonies on blood agar, and yellow colonies on mannitol salt agar (Carter et al., 1995). Under microscope, *S. aureus* appears as rounded seeds arranged in bunches, demonstrating its growth in various planes. *S. aureus* is the cause of a wide spectrum of illnesses whose symptoms range from superficial to fatal manifestations. It may colonize diverse sites on both human and animal body surfaces due to its commensal as well as opportunistic characteristics. *S. aureus* is a common inhabitant of the skin, mucosa, urinary tract, gastrointestinal tract, and, in particular, the anterior nares of the respiratory tract (Cuny et al., 2010). *S. aureus* has the ability to produce a wide variety of virulence substances such as various types of proteins, enzymes, toxins, and other substances responsible for high pathogenicity. *S. aureus* produces fibronectin-binding protein and protein A, both of which contribute to the bacterium’s ability to adhere to and colonize cell surfaces. The type of toxins produced by *S. aureus* are alpha, beta, gamma hemolysins, Panton-Valentine leukocidin (PVL) toxins, exotoxins, and enterotoxins. These all aid in the spread of *S. aureus* infection, which can cause severe blood stream and necrotizing infections in individuals (Gillet et al., 2002). This review article will cover the following aspects related to methicillin resistant strain of *S. aureus*; prevalence, transmission, pathogenesis, diseases, treatment, and prevention.

## 2. Emergence and types of MRSA

First time this bacteria was isolated from pus sample and given the name of “*S. aureus*” in 1881 (Ogston, 1881). Before the availability of penicillin, which was discovered by Radetsky (1996), an increased number of deaths were reported due to *S. aureus* infection that reached 90% case fatality rate which persisted up-to 19th century (Jevons, 1961). Later on production of β-lactamase enzyme by *S. aureus* makes the penicillin useless due to hydrolyzes of β-lactam ring of penicillin and *S. aureus* become resistant to penicillin soon after its discovery. Then, another antibiotic with the name of methicillin were discovered in 1950 which also found effective against *S. aureus* for long time. Unfortunately, the bacteria also acquired a significant resistance to this antibiotic and makes it ineffective anymore. The resistance to this antibiotic was reported at increased percentage which was name as methicillin resistant strain of *S. aureus* (MRSA). The molecular basis of MRSA was originated from a large mobile genetic element known as the staphylococcal cassette chromosome mec (SCC mec) genes such as *mecA* gene which are obtained by methicillin susceptible *S. aureus* (MSSA) through horizontal gene transfer among bacteria. The resistance was exhibited to all β-lactam antibiotics due to production of penicillin binding protein (PBP-2α) encoded by *mecA* gene (Vengust et al., 2006). In 1961, a study was conducted which noted among 50 staphylococci samples, 18 were found resistant to methicillin indicating high percentage (36.0%) of MRSA. These isolates were discovered to have the potential to keep both coagulase and hemolytic activity. MRSA detection was difficult in the early years of its discovery because methicillin resistance in *S. aureus* was diverse among different isolates and in a study, 5444 *S. aureus* samples were tested and only 3 isolates were diagnosed as MRSA (Barrett et al., 1968). As a result, heterogeneous strains mostly consist of bacterial cells that are both highly resistant and susceptible to methicillin. However, the addition of sugar or sodium chloride (NaCl) to the culture medium may promote the expression of phenotypic resistance in presence of β-lactam antibiotics (Datta et al., 2011).

From the long time, MRSA is considered as prototype of MDR and nosocomial pathogens which cause infection in hospitals and other healthcare settings. In the past three decades, the percentage of MRSA infections has significantly risen, and new strain of MRSA known as healthcare-associated (HA-MRSA) has spread and become endemic throughout industrialized nations as the leading cause of life-threatening infections like pneumonia, skin and bloodstream infections (Diekema and Pfaller, 2001). According to a US study, *S. aureus* infections are responsible for seven million hospitalizations of humans in the country, demonstrating the significant loss caused by hospital-acquired MRSA (HA-MRSA). The estimated yearly cost of these infections is $2.7 million, a considerable loss of 12,000 annual deaths, and sets the country’s economy at financial stress of over $9.5 billion (Noskin et al., 2005). First, MRSA was primarily associated with healthcare settings, and the risk factors that contributed to its spread were well-known (Chambers, 2001). A new type of MRSA rapidly emerged toward the end of the 1990s in the community and was known as community-acquired (CA-MRSA), with very high level of pathogenicity and the potential to spread, making it capable of infecting young as well as healthy individuals (DeLeo et al., 2010). Recently, MRSA also reported as frequent colonizer among animals due to extensive and frequent use of antibiotics in animal production. This new MRSA strain called as livestock associated MRSA (LA-MRSA) is primarily identified in food animals such as pigs, cattle, sheep, and goat with having extensive zoonotic potential (Pantosti, 2012). These cases of MRSA infection in humans and animals show how animals can serve as a source for the transmission of the disease, thus posing a serious threat to the population (Cefai et al., 1994). Rising public concern over MRSA has led to monitoring recommendations, such as information on the prevalence of the infection in healthy dogs, cats, and humans (Noskin et al., 2005).

## 3. Prevalence of MRSA

The global emergence and transmission of MRSA is one the most important aspect in the epidemiology of MRSA. The spread of all types of MRSA have been reported from many countries as listed in Table 1. The spread of MRSA is known to occur by one of the two ways which are either spread of existing clones among humans, animals, from animals to humans or humans to animals and acquisition of SSC*mec* element through horizontal genes transfer (Lee et al., 2018). Currently, MRSA is known to be more endemic in hospital settings and is among the major nosocomial pathogens. According to the statement of CDC, MRSA is known to be a major threat to public health because of its increasing prevalence in hospitals, community and animals, transmission between humans and animals, infection rates, resistance, and therapeutic issues (Ferri et al., 2017). On an average, it was estimated the annual health cost due to MRSA infections accounts 3 billion dollars. CA-MRSA has been also emerging as a principle pathogen from the recent years. It is noted MRSA mostly causes skin and soft tissue infection leading to bacteremia which lead to higher mortality rates ranging from 15 to 60% (Lee et al., 2013).

**TABLE 1:** Prevalence of livestock associated MRSA (LA-MRSA), healthcare-associated MRSA (HA-MRSA), and community-acquired MRSA (CA-MRSA) in different countries.

Sr. No	Year of study	Prevalence	Sample size	Sample sources	Country	References
**Livestock associated MRSA (LA-MRSA)**						
	2021	24.59%	787	Bovine milk samples	Pakistan	Lodhi et al., 2021
	2021	15.6%	200	Goat milk samples	Pakistan	Muzammil et al., 2021
	2020	30.0%	100	Tracheal and nasal samples of quails	Portuguese	Silva et al., 2021
	2020	30.43% in cats, 33.91% dogs, 25% in humans, and 50% in environment	384	Swab samples from cats, dogs, and environment	Pakistan	Shoaib et al., 2020
	2018	38.6%	95	Dairy cattle milk and nasal samples	Malaysia	Aklilu and Chia, 2020
	2017	34.0%	900	Bovine milk samples	Pakistan	Aqib et al., 2017
	2014	47.6%	450	Cow milk samples	China	Pu et al., 2014
	2013	6.3% from milk samples, 4.7% from hand and nose samples, 1.2% from farm environment	1146	559 milk samples, 86 hand and nose samples, 501 farm environment samples	Korea	Lim et al., 2013
	2012	71.5%	500	Swab samples of turkeys	Germany	Richter et al., 2012
	2011	16.7%	280	Cattle milk samples	Germany	Spohr et al., 2011
	2011	78% farm level 28% animal level	2151	Nasal samples of veal calves	Netherlands	Bos et al., 2012
	2009	1.41%	142	Cattle milk samples	Switzerland	Huber et al., 2010
	2009	2.9% in pigs and 1.6% in calves	1100	Nasal swabs from pigs and calves	Switzerland	Huber et al., 2010
	2007	0.4%	595	Cattle milk samples	Hungary	Juhász-Kaszanyitzky et al., 2007
**Hospital acquired MRSA (HA-MRSA)**						
	2008–2016	38.9%	318	Nose, ear, blood, pus, wounds, abscesses, eyes, genital	Norway	Enger et al., 2022
	2015–2017	45.0% (658/1466)	1466	Hospital patients	China	Chen et al., 2022
	2017–2021	26.0%	76	Ear, rectal swabs, oropharyngeal, nose, skin, wound, conjunctiva, bone, urine, synovial and peritoneal fluid, lymph node	Italy	La Vecchia et al., 2022
	2011–2019	35.1%	210	Inpatients and outpatients	Japan	Hosaka et al., 2022
	2018–2019	21.0%	164	Blood and soft tissues	Japan	Ogura et al., 2022
	2014–2020	23.4%	565	Blood, pus, wound exudate, sputum	China	Wang et al., 2022
	2020	15.1%	456	Blood	Australia	Coombs et al., 2022
	2021	34.8%	295	Urine, sputum, wound swabs, nasal swabs, fomites	Nigeria	Ugwu et al., 2022
	2019–2020	55.7%	97	Wound, pus, CSF, blood, skin lesions, sputum, joint fluid, ear, nose, throat	Iran	Tabandeh et al., 2022
	2021	19.1%	47	Nose, pharynx, and mobile phone	Mexico	Hamdan-Partida et al., 2022
	2020	43.44%	200	Wound, nose, cerebrospinal fluid of patients	Pakistan	ur Rehman et al., 2020
	2019	50%	742	children	Uganda	Kateete et al., 2019
	2017	52%	180	Human blood	Pakistan	Siddiqui et al., 2017
	2016	4.6%	726	Health care workers	Germany	Sassmannshausen et al., 2016
	2013	58.4%	1487	Humans	Portugal	Tavares et al., 2013
	2009	46.0%	6743	Humans	India	Arora et al., 2010
	2004	25.0%	758	Human patients	Texas	Davis et al., 2004
**Community acquired MRSA (CA-MRSA)**						
	2008–2016	61.0%	318	Nose, ear, blood, pus, wounds, abscesses, eyes, genital	Norway	Enger et al., 2022
	2015–2017	24.0% (105/434)	434	Community settings	China	Chen et al., 2022
	2018–2019	79.0%	164	Blood and soft tissues	Japan	Ogura et al., 2022
	2019–2020	16.0%	25	Nasal swabs	Egypt	Mostafa et al., 2022
		31.2%	565	Blood, pus, wound exudate, sputum,		Wang et al., 2022
	2020	84.9%	456	Blood	Australia	Coombs et al., 2022
	2021	28.7%	295	Urine, sputum, wound swabs, nasal swabs, fomites	Nigeria	Ugwu et al., 2022
	2019–2020	44.3%	97	Wound, pus, CSF, blood, skin lesions, sputum, joint fluid, ear, nose, throat	Iran	Tabandeh et al., 2022
	2018	23.5%	152	Nasal swabs	Pacific Asia	Wong et al., 2018
	2017	2.8%	404	Pig ear swab	China	Bi et al., 2018
	2017	1.7%	753	Nasal sample	China	Bi et al., 2018
	2012	64.7%	178	Pus sample	India	Alvarez-Uria and Reddy, 2012
	2009	38.5%	120	Dialysis patients	United Kingdom	Johnson et al., 2009
	2005	12.82%	14253	Nasal and inguinal swabs	Switzerland	Harbarth et al., 2005
	2005	7.3%	726	Anterior nares culture	Atlanta Georgia	Hidron et al., 2005

**FIGURE 1:**
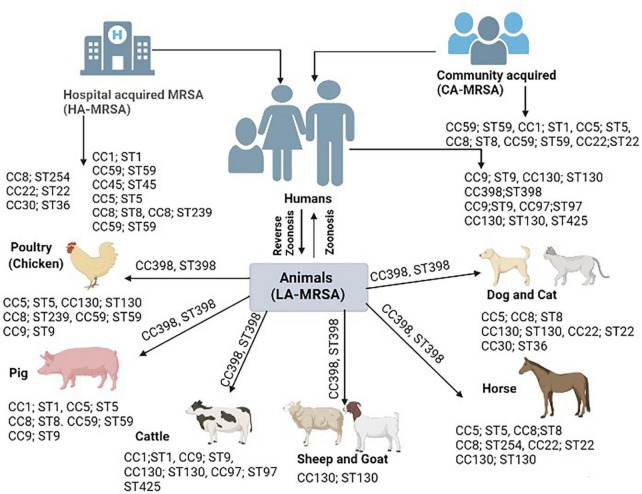
Bovine-adapted clonal complexes *Staphylococcus aureus* (*S. aureus* CCs) appear to have derived from human CCs and acquired bovine affinities through a series of spill-over events that resulted in the acquisition of various mobile genetic elements (MGEs). Several hosts have a high prevalence of the CC398 lineage. This lineage seems to have started in humans *via* reverse zoonosis, then it spread to pigs, then it returned to humans *via* pig zoonosis, and ultimately it spread to other species.

Recent trend in the prevalence of HA-MRSA was noted varying among the countries for example it was noted higher 58.4% from Portugal in 2013 (Tavares et al., 2013), 46% from India in 2009 (Arora et al., 2010), 52% from Pakistan in 2017 (Siddiqui et al., 2017), 45% from China from 2015 to 2017 (Chen et al., 2022), and 38.9% from Norway from 2008 to 2016 (Enger et al., 2022). However, with the increasing prevalence of HA-MRSA in different countries, MRSA prevalence also noted lower in many such as 4.6% from Germany (Sassmannshausen et al., 2016), 25% from Texas (Davis et al., 2004), 19.1% from Mexico (Hamdan-Partida et al., 2022), 15.1% from Australia (Coombs et al., 2022), and 26% from Italy (La Vecchia et al., 2022) are summarized in Table 1. Similar increasing and decreasing trend of MRSA prevalence from different countries also noted for CA-MRSA such as 79% from Japan (Ogura et al., 2022), 84.9% from Australia (Coombs et al., 2022), 64.7% from India (Alvarez-Uria and Reddy, 2012), 61% from Norway (Enger et al., 2022), and 44.3% from Iran (Tabandeh et al., 2022) with lower prevalence from Egypt (16%) (Mostafa et al., 2022), China [1.7% (Bi et al., 2018); 24% (Chen et al., 2022)], 7.3% from Gerorgia (Hidron et al., 2005), and 12.8% from Switzerland (Harbarth et al., 2005). This decline in the prevalence of HA-MRSA and CA-MRSA may be linked with better implementation national prevention measures. This increase and decline in the prevalence of HA−MRSA and CA-MRSA may be linked with increasing acquisition of LA-MRSA from animal reservoirs to humans especially from food and companion animals. The predominant LA-MRSA strain which is also identified from human MRSA isolates belong to CC398 as illustrated in Figure 1. However, person to person transmission of LA-MRSA found to be uncommon. The prevalence of LA-MRSA from different studies also noted higher from different countries as mentioned in Table 1. Recent studies conducted in Pakistan detected 15.6% LA-MRSA from goat milk (Muzammil et al., 2021), 24.5% from cow milk (Lodhi et al., 2021), 30.4% from cats (Shoaib et al., 2020), and 33.9% from dogs (Shoaib et al., 2020). Another study conducted in Malaysia in 2018 detected 38.6% MRSA from cow milk (Aklilu and Chia, 2020). Furthermore, two studies from Switzerland reported 1.41% from cow milk (Huber et al., 2010), 2.9% from pig nasal swabs (Huber et al., 2010), and 1.6% from calf nasal swabs (Huber et al., 2010) (summarized in Table 1).

## 4. MRSA transmission between humans and animals

Transmission of MRSA among different hosts is primarily known to happen by physical contact with source. The capability of transfer of MRSA among different host species including humans and animals is the characteristic feature of MRSA lineages. HA-MRSA is primarily acquired from hospital settings such as contaminated instruments, bedding, doors, and equipment’s while CA-MRSA is primarily acquired by physical contact with infected or healthy person as *S. aureus* is a commensal bacterial in the nares of healthy individuals. LA-MRSA transmission to humans when the individual has physical contact with animal and environment (Pantosti, 2012). Firstly, LA-MRSA was restricted only to animals until 1961 before the Hungarian cow was reported to be the source of LA-MRSA transfer to its caretaker by testing throat swabs (Cefai et al., 1994). This was the first report of MRSA transmission from animal to human which proved the ability of MRSA horizontal transmission among animals and humans. Later on a number of reports were published by various authors from different regions of world from different animal’s species such as poultry, pigs, cattle, sheep and goat, equines, and companion animals. These reports noted number of clonal complexes (CCs) such as CC5, CC8, CC9, CC59, CC1, CC30, CC45, CC22, CC130, CC97, and CC398 with multi-locus sequence types (STs) were found similar among human isolated MRSA strains and animal isolated MRSA strains. On the other hand, various HA-MRSA and CA-MRSA strains are also found similar to other LA-MRSA strains which are elaborated in Figure 1. A human clone ST1 was found in animals and is responsible of causing mastitis in bovines (Grundmann et al., 2010). Similarly, animals clone CC398 and lineage ST398 found among humans’ which cause infections similar to HA-MRSA and CA-MRSA (Witte et al., 2007). Moreover, a worldwide clone of poultry ST5 also found among humans working at poultry farms (Lowder et al., 2009). Similarly, a clone of small ruminants CC130; ST 130 also recovered from humans (Guinane et al., 2010). The MRSA transmission from companion animals to humans is also well-documented in various studies for example a study conducted in USA and Canada documented 18% carriage rate of MRSA among the owners of companion animals (Faires et al., 2009). Another study in UK in nursing home documented similar strain in patient, hospital staff and nursing cat (Scott et al., 1988). Similarly, a study conducted at veterinary hospital detected transmission of MRSA lineage ST22 from infected dogs to veterinary staff (Baptiste et al., 2005). However, pets also found to be colonized with human PVL positive CA-MRSA strain by a household member in Netherland (Van Duijkeren et al., 2005). The risk of getting MRSA in animal caretakers (1.7%) was higher compared to those who were not exposed to animals (Wulf et al., 2006). For 13 months, eleven horse patients were hospitalized at the veterinary hospital for various diagnoses and surgical procedures. After procedures, a strain of MRSA was identified. MRSA strain was also isolated from 3 of 5 and with 1 person found to be colonized with 2 biotypes of MRSA. The isolates of human MRSA appeared to be identical to those of horses. The results showed that the isolates of horses and humans are members of a very close group and, apparently come from a common source. According to the pattern associated with the infection, a special mode of transmission was still unknown, but it was assumed that the main cause of the infection is the staff of the veterinary hospital (Seguin et al., 1999).

A number of risk factors also play a significant role in the spread of CA-MRSA and HA-MRSA infections. Important risk variables for cellulitis were overweight, the existence of abscesses, and head-and-neck sores relative to infections produced by other microorganisms according to an analysis of individuals with the condition. The presence of abscesses and obesity were additional important risk variables for MRSA dermatitis (Khawcharoenporn et al., 2010). Significant correlations between MRSA colonization, skin infection in the previous 3 months, sharing soap, and MRSA skin and soft tissue infection (SSTI) against no SSTI were found, college education, knowing about “staph” before, taking bath daily, and the previous contact with a health care worker (Haysom et al., 2018). Further research compared those with MRSA to those with MSSA to identify risk variables for MRSA colonization. According to research, there are a variety of meaningful risk factors for MRSA infection, such as the involvement of family members under the 7 years of age, a smoking habit, and the consumption of antibiotics the year before. Additional characteristics including age, sex, married status, chronically sick patients, education, taking a daily shower, and family income, however, were not meaningful risk factors for MRSA colonization (Wang et al., 2009).

Except these risk factors in humans, a study also highlighted the risk factors associated with LA-MRSA mammary infection in dairy animals. Among those risk factors are animal parity number, age, feeding status, body condition score, udder hygiene, hand or machine hygiene while milking were important factors associated with this infection while milking frequency was found non-significant risk factor for LA-MRSA infection (Aqib et al., 2017). Another study conducted by Shoaib et al. (2020) highlighted the risk factors associated with transmission of LA-MRSA from companion animals. Among those risk factors, animal health status, infection on body, long term antibiotic therapy, veterinarians, pet access to bedroom were found significant risk factors in transmitting MRSA to humans while the size of dog, owner’s sex, and sample site were found to be non-significant risk factors associated with MRSA transmission. Another study conducted by Mulders et al. (2010) highlighted the possible risk factors of MRSA transmission from poultry to humans are farm workers, individuals having contact with live birds at slaughterhouse, type of slaughtering method, and slaughtering environment are significantly correlated with higher carriage of MRSA among humans.

## 5. MRSA pathophysiology

**FIGURE 2:**
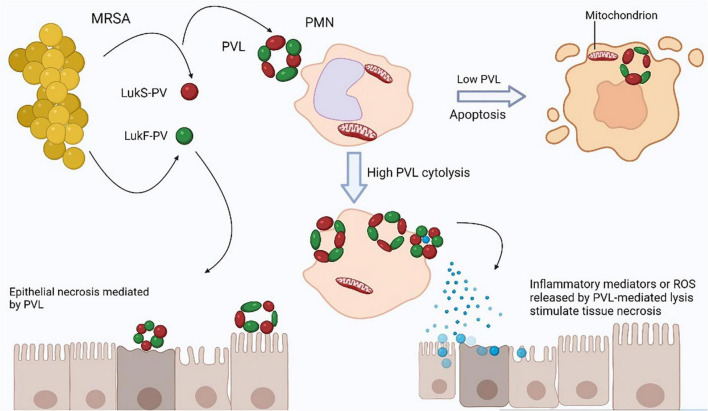
Tissue necrosis is caused by Panton-Valentine leukocidin (PVL). The two PVL components secreted by *Staphylococcus aureus*, LukS-PV, and LukF-PV, collectively form a pore-forming heptamer on the membranes of polymorphonuclear leukocytes (PMNs). Low PVL concentrations cause polymorphonuclear leukocytes (PMN) apoptosis through direct binding to mitochondrial membranes, whereas high PVL concentrations cause PMN lysis (Genestier et al., 2005). From lysed PMNs, reactive oxygen species (ROS) can cause tissue necrosis. Furthermore, the release of granules from PMNs that have been lysed may cause an inflammatory response that leads to tissue necrosis. PVL is unlikely to cause direct necrosis of epithelial cells.

*Staphylococcus aureus* is a pathogenic and commensal bacterium that normally lives in the anterior nares of both humans and animals as well as in axillae, groin, and gastrointestinal tract are sites where it can also colonize. The major steps in pathogenesis of infection are colonization, virulence, initiation of infection, abscess formation, systemic infection, regulation and adaptation with the help of number of virulence factors. Colonization enhances the risk of bacterial infection when the host’s defenses are compromised either by physical disruption or other diseases (Wertheim et al., 2005). As MRSA is the methicillin-resistant strain of *S. aureus*, the *S. aureus* by self-contain a number of potential virulence factors which includes several surface proteins called “microbial surface components recognizing adhesive matrix molecules” (MSCRAMMs), which bind to fibrinogen, fibronectin, and collagen fibers of host cells to attack the host tissues. These factors may lead to infections of prosthetics, bones, joints, and endovascular system (Menzies, 2003). The ability of *S. aureus* to produce biofilm on both prosthetic surfaces and the host allows it to adhere to those surfaces by evading the effect of antimicrobials and host immune system. *S. aureus* also have the ability of produce small colony variants (SCVs) which have ability to cause persistent and recurrent infections. *S. aureus* also contain the anti-phagocytic microcapsule (type 5 or 8) which act as a primary defense mechanism. Moreover, due to internaction of Zwitter ionic capsule with Fc region of an immunoglobulin, the MSCRAMM protein A facilitate the protection of *S. aureus* from opsonization (Gordon and Lowy, 2008). Except these *S. aureus* contain a number of virulence factors such as adhesion proteins, chemotaxis inhibitory proteins, various enzymes such as proteases, lipases, hyaluronidase, staphylokinase, catalase, nucleases, lipases, coagulase, catalase, proteases, collagenases, β-lactamases, and elastases which help the *S. aureus* in causing infection in host. Except these virulence factors, MRSA also contain different mobile genetic elements (MGEs) in different animals’ species as listed in Table 2 that also aid and enhance its pathogenicity. Besides these, *S. aureus* produces various type of toxins such as exotoxins, enterotoxins, TSST-1, hemolysin toxins and PVL toxins as illustrated in Figure 2. Additionally, certain strains of *S. aureus* release superantigens that can also cause infections such as food poisoning and toxic shock syndrome (TSS) (Dings et al., 2000). Normal expression of *S. aureus* virulence factors plays a significant role in pathogenesis. Virulence factors express only according to the requirement of the bacterium to decrease unnecessary metabolic demands. Although secreted proteins like toxins are generated during the stationary phase, MSCRAMMs typically express during logarithmic growth phase. Early MSCRAMM protein expression helps in the initial colonization of tissue sites, while late toxin production helps in the dissemination of infection into the bloodstream. Mainly the *S. aureus* pathogenicity is regulated by the quorum-sensing accessory gene regulator (AGR) (Gordon and Lowy, 2008). *S. aureus*, in short, has a wide range of ways to cause disease and evade host defenses. However, the existence of some virulence factors is independent of the genomic structure and are related to clonal type (Peacock et al., 2002).

**TABLE 2:** Methicillin resistant strain of *Staphylococcus aureus* (MRSA) mobile genetic elements (MGEs) associated host determinants in different species.

Disease	Host	MGEs	MGE-linked host determinants	References
Mastitis, skin infections	Ruminants	SaPIbov, enterotoxin gene cluster, SaPIbov4, Non-mec SCC, SCC-*mecC*	Sec, Seg, Seo, Sel, Sei, Sem, Sen, TSST-1, ssl07, ssl08, vWbp, and LPXTG surface protein	Viana et al., 2010; Wilson et al., 2011; Resch et al., 2013; Bar-Gal et al., 2015
Neonatal septicaemia, skin infections	Swine	Pathogenicity islands (SaPI-S0385) and plasmids	SSC*mec*, SaPI5, SaPlbov1, vWbp, resistance to heavy metals	Schijffelen et al., 2010; Richardson et al., 2018
Skin, thoracic and joint or bursal infections	Equine	ΦSaeq1, SaPIeq1	Immune modulators, Scn gene, *luk*PQ genes, vWbp	Viana et al., 2010; Garzoni and Kelley, 2011; Koop et al., 2017; De Jong et al., 2018
Skin and soft tissue infections	Pets	Plasmid SAP078A, SCC*mec* type IV, bacteriophage Φ2, Φ3, Φ6, rep_10_ plasmid, SaPI	Replication genes (rep_5_, rep_22_), heavy metal resistance genes (copB, arsR, cadC, arsA, mco, and cadA), host immune evasion genes (scn, sak, and chp),	Loeffler et al., 2013
Pododermatitis	Poultry	ΦAvβ, pAvX, pAvY, pC221, ΦAv1, SaPIAv, pUB112	ornithine cyclodeaminase, protease, ear-like proteins (ear pathogenicity islands such as SaPI1, 3, 5 and SaPImw2, Thiol protease ScpA, Lysophospholipase, Tetracycline and Chloramphenicol resistance	Ehrlich, 1977; Lowder et al., 2009; Murray et al., 2017
Lung infection, Bloodstream infections, Endocarditis, Osteomyelitis,	Human	ΦSa3 (β-hemolysin converting phage)	(clfB, isdA, fnbA, atlA, eap), genes involved in Wall Teichoic Acids (WTA) biosynthesis (tagO and tarK), cell surface dynamics/remodeling enzymes (sceD, oatA, atlA), immune-modulatory factors, TSST-1, PVL toxins and staphylokinase	Manders, 1998; Foster, 2002; Richardson et al., 2018

Hospital acquired methicillin-sensitive *S. aureus* (MSSA) is less dangerous pathogen than HA-MRSA, which increases the pathogenicity and mortality. Nonetheless, the specific pathogenicity mechanism is unknown. However, it is thought that the PBP2-α protein, which is associated with β-lactam antibiotic resistance and expressed by the *mecA* gene, directly contributes to immunopathology during MRSA infection. Poor peptidoglycan cross-linking with β-lactam antibiotic caused by PBP2-α results in increased survival of MRSA strains as compared to MSSA (Yao et al., 2010). Improved immune system evasion and *S. aureus*-exclusive toxin synthesis all contribute to CA-MRSA strains’ enhanced virulence. Researchers have found that *S. aureus*’s PVL protein has dermonecrotic and leukocyte-lysing properties that lead to increased pathogenicity of CA-MRSA strains (Chini et al., 2006). Further investigations are required, since studies claim that the relationship between PVL and CA-MRSA virulence is complex (Wardenburg et al., 2007). Another study conducted by Wang et al. (2007) also showed that phenol-soluble modulin proteins which promote inflammation and impair the function of neutrophils in bacteremia patient and mice models were more abundant in CA-MRSA strains than HA-MRSA strains. The emergence of LA-MRSA and its transmission to humans and reports of humans strains in animals further increases the pathogenicity of MRSA as now MRSA have diversity of host species which results in genomic modification and increases the antibiotic resistance. SCC*mec* cassettes, particularly SCC*mec* IVa and SCC*mec* V, are present in LA-MRSA while other cassettes, such as SCC*mec* type XI, which includes *mecC*, have also been reported (Voss et al., 2005). According to several studies, the LA-MRSA CC398 misplaced human-associated virulence factors like exfoliative toxins and acquired the antibiotic resistance genes like *mecA*, *tetM*, TSS toxin I, and PVL genes (Ballhausen et al., 2017). The staphylococcal protein A gene (spa) in CC398 are also currently reported which aid in MRSA pathogenicity (Peeters et al., 2015).

## 6. MRSA infections in humans

Methicillin resistant strain of *S. aureus* (MRSA) is an emerging pathogen that can cause mild to serious infections in both animals and humans. Among humans, it mostly causes mild to life deadly infections such as skin and soft tissue infections which are staphylococcal scalded skin syndrome (SSSS), pustules, impetigo contagiosa, abscesses, and papules while deadly infections include TSS, pneumonia, or newborn TSS-like exanthematous disease in humans (Takahashi et al., 1998). Among the 100,000 cases of MRSA infections per year, 20% of the patients died (Klevens et al., 2007). Previously, CA-MRSA and HA-MRSA were two major types of developing infections in humans. HA-MRSA infections are seen in athletes, children, and in hospitalized individuals while CA-MRSA most offenly causes various types of SSTIs which range from mild such as fruncles, impetigo to deadly such as necrotizing fascilitis, and pneumonia infections. CA-MRSA causes less severe infections in animals and humans as compared to HA-MRSA (David and Daum, 2010).

### 6.1. Hospital-acquired MRSA

Significant risk factors associated with HA-MRSA infections are aged and immunosuppressive patients due to extensive usage of broad-spectrum antibiotics for a long time (Kurkowski, 2007). HA-MRSA shows resistance to almost all β-lactam drugs which is the reason for its spread among hospital-acquired infections (Lindsay, 2013). Compared to HA-MRSA, CA-MRSA may be found in healthy people and its prevalence may be high among the people working at day care centers, prisons, players, and among military personnel because they are living in close contact with each other (Daum, 2007). HA-MRSA being a multidrug-resistant organism causes many healthcare-associated infections in children and adults. The infection rate is high in people that are immune compromised and in patients having cuts on the skin that may a source of spread of infection (Köck et al., 2010). HA-MRSA clones are dominant bacterial clones that are the major cause of these types of infections in humans and animals. These clones are distributed differently in their geographical areas. HA-MRSA epidemics start in the 1980s or 1990s and the major reason behind this was due to the development of novel clones of MRSA (Chambers and DeLeo, 2009). These clones circulate quickly among hospitals and lead to an increase in the death and morbidity rate. The important HA-MRSA clones include CC, spa type, sequence type (ST), PAGE type, and most simply by their lineage type. Among the CC includes CC30, CC5, CC45, CC8, and sequence type 239 (ST239) (Okuma et al., 2002; McDougal et al., 2003; Naimi et al., 2003; Klevens et al., 2007).

### 6.2. Community-acquired MRSA

**FIGURE 3:**
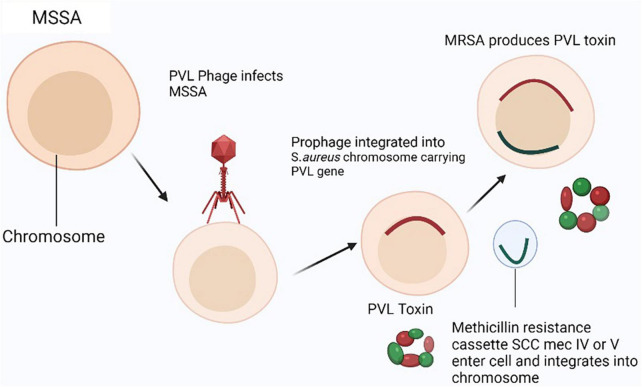
Panton-Valentine leukocidin (PVL) producing community-acquired–MRSA (CA-MRSA) model: In MSSA strain, two genes (pvl) encoding the methicillin-resistant phage virus (PVL) are infected and lysed by the phage (phiSLT) (Staphylococcal Leukocytolytic Toxin). Then, a horizontal transfer of a methicillin resistance cassette (SCCmec IV, V, or VT) carrying the mecA gene into the pvl-positive methicillin susceptible *Staphylococcus aureus* (MSSA) strain allows it to incorporate into the genome somewhere other than the phiSLT (Staphylococcal Leukocytolytic Toxin) integration site.

Community-acquired MRSA (CA-MRSA) causes infections on several body parts, most commonly skin and soft tissues, but also in lungs, bone, joints, bloodstream, surgical sites, and urinary tract (Dantes et al., 2013). Although, CA-MRSA is not limited to the skin and soft tissues but also the major cause of septicemia and necrotizing pneumonia. CA-MRSA also causes bacteremia that’s a complication that will lead to endocarditis and osteoarticular infections (Alzomor et al., 2017). CA-MRSA is becoming a global issue among infants, children, and adolescents (Stankovic and Mahajan, 2006). Researchers in Japan using techniques like agr typing, spa typing, coagulase typing, PCR assay for virulence genes, SCC*mec* typing, and multi-locus sequence typing (MLST) characterized the PVL-positive CA-MRSA in children in 2003 and similar strain was noted in athletes having cutaneous abscess (Takizawa et al., 2005). A rise in CA-MRSA infections was noted up-to 29.8%, and left 70.2% was due to unknown risk factors in Saudi Children Hospital. A study conducted in King Fahad Medical City, Riyadh, Saudi Arabia from 2005 to 2008 among outpatient children showed that 29.8% of cases were positive for CA-MRSA while the other 70.2% were due to unknown risk factors (Mermel et al., 2009). Initially, it was believed that CA-MRSA was a nosocomial strain that had been transmitted from hospitals to the population. However, unlike the HA-MRSA strains often identified in healthcare settings, CA-MRSA strains are paradoxically sensitive to non-β-lactam antimicrobials and showed clinical symptoms more resembling those of MSSA strains (Herold et al., 1998). The genetic lineage, genetic make-up of the methicillin resistance genes, and existence of PVL are the three main attributes that differentiate a CA-MRSA strain from a HA-MRSA strain. The current evidence indicates several distinct MSSA ancestral clones that are circulating in the world are incorporated by SCC*mec* especially SCC*mec* IV in CA-MRSA strains through gene transfer mechanism (Figure 3; Enright et al., 2002).

## 7. LA-MRSA infections in food and companion animals

Although the spread of MRSA infections in food and companion animals was initially thought to be slower, it is now becoming a serious problem for food animals and food industries too. LA-MRSA is an important cause of mastitis in cows and buffaloes resulting in a decrease or no milk production (Javed et al., 2021). LA-MRSA also causes the infections in poultry such as comb necrosis, chondronecrosis, and septic conditions (Fluit, 2012). Almost all the companion animals like dogs, cats, and horses, are potential sources of LA-MRSA transmission to humans having direct or indirect contact with these animals. Besides mammals, LA-MRSA colonization in foxes, roes, rabbits, wild boars, and wild animals (e.g., pigeons, pheasants, ducks, buzzards, gulls, and rocks) has also been reported (Smith et al., 2009).

### 7.1. MRSA infection in food animals

**FIGURE 4:**
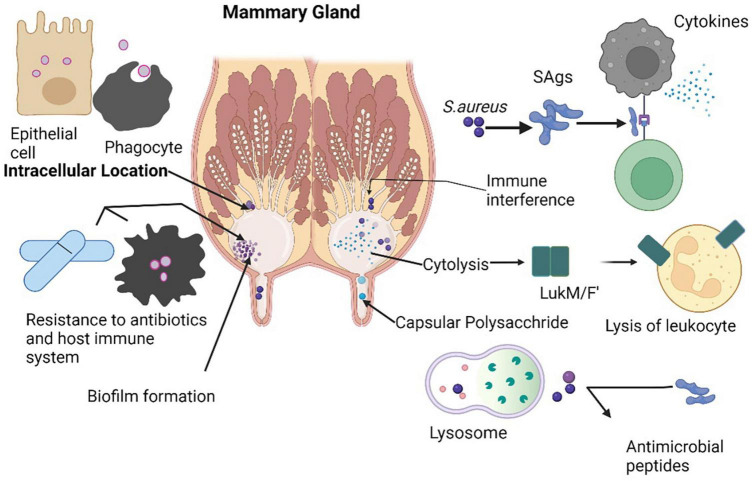
Different tactics used by *S. aureus* to survive inside mammary glands to cause infection.

Mastitis is an important disease of dairy animals, which is linked to the maximum use of antibiotics responsible for huge economic losses. *S. aureus* is a chief pathogen of mastitis among all other causative agents throughout the world. LA-MRSA is also an important cause of pustular dermatitis in their milkers (Grinberg et al., 2004). Contrarily, all bovine MRSA clones are infrequently seen in dairy cows, responsible of subclinical mastitis in cattle (Aqib et al., 2018b; Abdeen et al., 2021). The first time MRSA was detected in Cattle in Belgium in 1972 in milk samples which thought to be spread from milker hands through contamination (Lee, 2003). MRSA infection of mammary gland in cattle leads to a decrease in milk production and may cause cessation of milk from mammary glands in severe cases which pose the dairy industry a big economic loss (Figure 4; Holden et al., 2013). Mammary gland inflammation results from cytotoxicity of *luk*MF9, a powerful virulence factor of LA-MRSA (Peton et al., 2014). *luk*MF9 has a high affinity for chemokine receptor CCR1 on bovine which results in significant inflammation and damage as a consequence of more accumulation of neutrophils in mammary gland (Fromageau et al., 2010; Vrieling et al., 2015). A tropical phage called wPV83, which is capable of hematogenous spread to *S. aureus*, carries the *luk*M and *luk*F genes (*luk*M-*luk*F-PV) which are closely associated with synthesis of *luk*MF9 (Yamada et al., 2005). A conformational change in the nucleotide of a gene produces a suppressor protein (*rot*) in *S. aureus* strains that block the activation of many toxin genes that exhibit large quantities of the *luk*MF9. Genetic studies have linked strains overexpressing *luk*MF9 to the lineage ST479 (Hoekstra et al., 2018). Superantigens (*SAgs*) are a class of bacterial toxins that stimulate the immune system and are released by staphylococcal species, particularly *S. aureus*. They have the potential to cause an unsustainable cytokine cascade (Tuffs et al., 2018). However, SAgs seem to perform a crucial function in bovine mastitis, and the majority of cattle *S. aureus* isolated have five or more genes encoding *SAgs*, although the precise function of *SAgs* in mastitis is yet unknown (Wilson et al., 2018). *SAgs* disrupt the human autoimmune reaction, which makes them potentially significant in chronic infections (Ferens and Bohach, 2000). By preventing immune-regulatory activation, the multiplication of T cells and the production of interleukins may stopped by SAgs that might reduce the efficiency of the immune system response (Tuffs et al., 2018). The cow susceptibility island SaPIbov is present in lineages that are related to cattle, including CC151 and CC133 (Wilson et al., 2018). Numerous toxins, including the toxic shock syndrome toxin 1 (TSST-1), the staphylococcal enterotoxin-like protein, and bovine staphylococcal enterotoxin C are also stimulated by SaPIbov (Fitzgerald et al., 2001).

Pigs in various studies exhibit higher than expected percentages of MRSA. Highly pathogenic strains are also rising in the pig population. In a study, 10% of samples were positive for the ST398 strain of MRSA, and the overall presence of MRSA among the three studied farms was considerably high. These 10% ST398 strains of MRSA were further found related to spa type, t1793 and t034 and they were exhibiting a high level of resistance to multiple antibiotics. This is another clue for the new emerging zoonotic strain of MRSA in Europe among the pig population (Hasman et al., 2010). Moreover, LA-MRSA ST398 is not only confined to pigs in Europe but it also spreads to Canada and USA (Khanna et al., 2008; Smith et al., 2009). Except ST398 strain, pigs also harbor other LA-MRSA strains such as ST1, ST97 and ST9 but at a lower frequency. A study conducted by Wagenaar et al. (2009) in China identified LA-MRSA ST9 lineage type colonization in pigs as well as workers. Another study was conducted by identified the human clone ST1 in pigs which indicating human to pig transmission of clone called as reverse zoonosis as depicted in Figure 1 (Monaco et al., 2010).

Methicillin resistant strain of *S. aureus* (MRSA) is a chief bacterium isolated from mastitis milk of goats which may infect individuals who are consuming contaminated milk. Contaminated milk may contain certain types of staphylococcal enterotoxins (SE), which is a major cause of food poisoning. All SE genes are capable of causing illnesses that are particular to their hosts, such as staphylococcal enterotoxin B causes infection in humans (Da Silva et al., 2005; Altaf et al., 2020). MRSA can also cause pyaemia which is a type of abscess which gain access to bloodstream and lead to sepsis (Webster and Mitchell, 1989). MRSA in sheep and goats is among the predominant cause of clinical and subclinical mastitis which leads to high somatic cell count in milk and make milk unfavorable to drink (Muzammil et al., 2021).

Livestock associated MRSA (LA-MRSA) has been also reported in cloaca and nares of healthy poultry birds. In poultry birds, it may cause pyoderma, omphalitis, urinary tract infection, arthritis, and otitis (Pickering et al., 2022). After performing different antimicrobial agents. MRSA was found spa type t011 and spa type t157. While ST398 is a new livestock-associated strain of MRSA that is also found in poultry (Nemati et al., 2008). Another investigation showed that all MRSA isolates of poultry origin were belonging to spa type t1456 (Persoons et al., 2009).

### 7.2. MRSA infection in companion animals

The companion animals mostly suffer skin and soft tissue infections predominately after surgical procedures. The United Kingdom observed 95/6519 MRSA-positive samples that were comprised of 24 cats, 69 dogs, 1 horse, and 1 rabbit (Boag et al., 2004). Molecular analysis of MRSA in dogs and cats using SCC *mec* typing and sequence typing of *ccrAB* gene (cassette chromosome recombinant gene AB), and established MRSA strains using MLST from cats and dogs similar to that of human SCC *mec* gene in staphylococci. A further cross-sectional study revealed the transmission of MRSA strains from humans to dogs especially MRSA strain ST239-III (Malik et al., 2006). A research finding at Irish Veterinary Hospital noted that 69.44% of canines, 22.22% of horses, and 2.78% of cats, rabbits, and seals were infected with MRSA (O’Mahony et al., 2005). Risk factors such as the site of infection, surgical history, medical history, intravenous catheterization, and use of previous antibiotics were inferred after 6 years of a controlled trial conducted in veterinary health care in the USA and Canada. The most prevalent sites of colonization were found skin and nares (Manian, 2003). MRSA also causes remarkable and life-threatening infections in horses and personals taking care of them. Horses may have septic arthritis, bacteremia, osteomyelitis, and inflammation of the skin and delicate tissues (Cuny et al., 2006), metritis, omphalitis, pneumonia, and infections related to catheters are a few examples of illnesses associated with implants. First case of MRSA in horses was noted in 1993 due to postoperative infections. Reverse zoonosis with CA-MRSA-5 (ST8) in horses has also been found with a wider range of antibiotic resistance, e.g., oxacillin, tetracycline, gentamycin, etc., (Weese et al., 2005).

## 8. Current and futuristic approaches to treat MRSA infections

Among individuals who have established MRSA infections, bacteremia that might result in mortality and could affect nearly 50% of the population (Nickerson et al., 2009). MRSA preventive measures and use of a recent antibiotic therapy alone and in combination may result in decrease MRSA infections (De Kraker et al., 2013). The ineffective disease management at the start of infection may be the reason of excessive death rate (Simor et al., 2016). Additionally, several virulence genes have been linked to increased mortality, such as acquisition of antibiotic resistance genes through horizontal gene transfer mechanisms are major cause of making antibiotics ineffective against MRSA infection (Albur et al., 2012). However, still there are antibiotics and futuristic approaches to treat MRSA infections which are discussed in further sections. The advent of several new antibiotics alone or in combination are available in the market, a hope for MRSA infected patients. Additionally, scientists now are switching from single-agent therapy to combination therapy, immunotherapy, and few latest alternative ways to combat MRSA infections such as phytochemicals, probiotics, nanoparticles, and bacteriophages as an antibiotic alternatives (Lee et al., 2016).

### 8.1. Antibiotics to treat MRSA infections

In comparison to MSSA infection, bacteremia caused by MRSA is more severe (Fowler et al., 2006). A prolonged duration of bacteremia may result in a more severe consequences (Fowler et al., 2003). A survey conducted in Australia noted that ceftaroline and cephalosporin resistance were found in 17% of MRSA cultures. Although several other new drugs have been approved for use, vancomycin remains the most effective (Abbott et al., 2015). Moreover, combining a β-lactam antibiotic with glycopeptide for example daptomycin to treat MRSA infections is recommended by the Spanish Society of Clinical Microbiology and Infectious Diseases (Gudiol et al., 2015). The mechanism of action of daptomycin somewhat different, it causes potassium and calcium ions to cross the plasma membrane, which causes apoptosis of the bacterial cell. Daptomycin inhibits the function of *fem* and *aux* genes, hence reducing the expression of the *mecA* gene. Daptomycin can reduce PBP-2α binding to its peptidoglycan moieties in the early phases of peptidoglycan synthesis (Rand and Houck, 2004). Daptomycin’s ability to bind to β-lactam antibiotics is improved when used in combination (Dhand et al., 2011). However, mutation at the gene level may makes the daptomycin a resistant drug against MRSA. The major genes involved in daptomycin resistance include multi-peptide resistance (*mprF*) and the regulatory gene *walKR* (also called *yycGF*). This is because of polymorphism in single nucleotide sequences (Jiang and Peleg, 2015). Another type of mutation e.g., point mutation in genes such as *rpoB* and *rpoC* also associated with the development of resistance against daptomycin (Peleg et al., 2012).

Vancomycin was once thought to be the most efficient antibiotic for treating MRSA-related severe infections. The suitability of vancomycin’s primary action is determined by gathering evidence of overall resistance, unachievable pharmacokinetic/pharmacodynamic (PK/PD) targets, and reduced effects (Holmes et al., 2012; Van Hal and Fowler, 2013). Vancomycin intermediate *S. aureus* (VISA) and hetero resistant (hVISA) are both commonly treated with glycopeptides. Vancomycin, therefore, begins to become resistant to them as well as losing sensitivity to glycopeptides, which causes the development of vancomycin-resistant *S. aureus* (VRSA). These isolates are more sensitive to other antibiotic classes, particularly β-lactam antibiotics, despite having the *mecA* gene. The “seesaw effect” refers to the sensitivity of MRSA isolates to anti-staphylococcal β-lactam antibiotics caused by higher minimum inhibitory concentrations (MICs) of daptomycin and vancomycin. Research on the synergistic effects between vancomycin and β-lactam antibiotics presents great potential (Werth et al., 2013). When the β-lactam seesaw effect and cross-resistance among glycopeptides, lipopeptides, and lipoglycopeptides against MRSA strains were examined, the important factors responsible for developing the cross-resistance to daptomycin, vancomycin, and dalbavancin is the change in membrane lipid composition such as fatty acyl and ultimately resulted in resistant strains. Abundance of long-chain fatty acyl peptidoglycans in membrane has a negative correlation with β-lactam sensitivity and a positive correlation with cross-resistance (Hines et al., 2020).

The combined effect of β-lactam antibiotics and vancomycin is found synergistic in *in vitro* studies when checked through antimicrobial sensitivity assays. The synergistic effect is highly linked with MIC of vancomycin. An *in vivo* trial was conducted on rabbits infected with VISA strain. The efficacy of two drugs, vancomycin and nafcillin, was checked to compare their effects. The results were ineffective with a single administration of drugs but give curative effects with combination therapy by reducing the magnitude of infection up to 4.52 log_10_ CFU/g. The extent of synergism was highly correlated with the MIC of vancomycin (Climo et al., 1999). Another *in vitro* study evaluated combinations of vancomycin and cephalosporin showed synergistic effects against MRSA isolates (Seibert et al., 1992). These isolates were also checked against the combination therapy of vancomycin and imipenem under the synergistic effect (Silva et al., 2011). Many more combinations have also been inquired such as rifampicin and gentamycin; daptomycin and rifampicin therapy against biofilm-producing MRSA isolates (Rosenberg Goldstein et al., 2012). These therapies proved better results by comparing the daptomycin and cloxacillin in a rat infected with MRSA and indicated better results against rifampicin-resistant MRSA infections.

The fifth generation cephalosporins are the most effective against MRSA infections than other antibiotic generations. The most widely utilized drugs in health care facilities among cephalosporins are ceftaroline and ceftobiprole. Ceftaroline is a drug that is licensed to treat skin associated infections and community-acquired pneumonia (CAP) (Purrello et al., 2016). To compare the efficacy of ceftobiprole, ceftriaxone, and linezolid, multi-center clinical studies were carried out in 2006–2007 among patients in hospitals who exposed to CAP. A multi-center FOCUS-1 study, conducted by a researcher in 2008–2009, investigated the effectiveness of ceftaroline and ceftriaxone against CAP. Nearly 168 locations from around the world were chosen. The modified intention to treat (mITT) and clinical evaluation (CE) rates of ceftaroline were found to be high, 86.6 and 83.3%, respectively while of ceftriaxone were 77.7% in mITT and 78.2% in CE (File et al., 2011). Many new approved and novel drugs are effective against MRSA infections, but further research is required to determine their efficacy at large scale. The next section discusses few of the most recent antimicrobials that are effective against MRSA infections (Thati et al., 2011).

#### 8.1.1. Oxazolidinones

A novel class of antibiotics known as oxazolidinones are effective against a variety of gram-positive bacteria, including vancomycin- and methicillin-resistant staphylococci. Oxazolidinone blocks protein synthesis by binding to the P-site of the 50S ribosome subunit. The development of oxazolidinone resistance with 23S rRNA does not influence oxazolidinone action when compared with the resistance of other protein synthesis blockers. Its application in surgical infections is made possible by its effective penetration in bone and infiltration into lungs, cerebrospinal fluid, and hematoma (Bozdogan and Appelbaum, 2004). Tedizolid, a brand-new drug among the oxazolidinones, has received approval for the standard 6-days treatment course for skin and soft tissue infections. Compared to linezolid, tedizolid is a drug that is more efficient and offers more benefits (Boucher et al., 2014; Flanagan et al., 2015). Tedizolid’s efficiency against chloramphenicol and florfenicol resistant (CFR) isolates harboring methyltransferase gene is also found (Flanagan et al., 2015). Novel oxazolidinone agent cadazolid is a potent agent against *Clostridium difficile* (Gerding et al., 2015), while radezolid is efficient against isolates of *S. aureus* that are resistant to linezolid (Lemaire et al., 2010).

#### 8.1.2. Tetracycline

This class of antibiotic inhibit the bacteria growth by inhibiting the protein synthesis. They attach to the 30S ribosomal subunit and prevent aminoacyl-tRNA from joining the translational mRNA complex, which inhibits the beginning of translation. In several researches, the drug tetracycline was found to bind to the rRNAs 16S and 23S (Chukwudi, 2016). The novel synthetic fluorocycline and eravacyline drugs are effective against infections caused by gram-positive and gram-negative bacteria, including MRSA. Eravacycline is four times more efficient than tigecycline for treating gram-positive bacteria (Zhanel et al., 2016). Omadacycline, an aminomethylcycline, is more efficient to treat CAP and acute bacterial skin and skin structural infections (ABSSSI) (Pfaller et al., 2017).

#### 8.1.3. Fluoroquinolones

Quinolones are among the most common antibacterial drugs used worldwide to treat a range of bacterial diseases in both animals and humans. These drugs are structurally known as quinolones because they have a quinoline ring in their structure. Quinolones and fluoroquinolones prevent bacteria from multiplying by inhibiting their DNA replication pathway. These antibiotics basically damage the bacteria chromosome by targeting the enzymes gyrase and topoisomerase IV (Aldred et al., 2014). The fluoroquinolone antibiotic, delafloxacin is an drug that is effective against both gram-positive and gram-negative bacteria because of its unique electrochemical characteristics such as being anion at physiological pH and uncharged at acidic pH (Lemaire et al., 2011). In 2011, a research trial was carried out in the USA to evaluate delafloxacin effectiveness in comparison to vancomycin and linezolid. These three drugs were found in following order with highest rates of cure, delafloxacin, linezolid, and vancomycin (Kingsley et al., 2015). Zabofloxacin is another fluoroquinolone drug showing high activity against gram-positive microorganisms, especially against respiratory tract infections due to *St. pneumoniae.* MIC_50_ of zabofloxacin against MRSA was noted high as compared to delafloxacin i.e., 2 mg/ml in and 0.125 mg/ml respectively. Except for these characteristics of zabofloxacin, it is less efficient against gram-negative organisms. Thirdly, nemonoxacin was found to be similar to zabofloxacin in its pattern of activity and its effects against CAP have been also investigated. Fourthly, avarofloxacin efficacy against MRSA is also comparable to that of delafloxacin (Van Bambeke, 2014).

#### 8.1.4. Lipoglycopeptides

Fatty acid chains linked to glycopeptides, a family of antibiotics known as lipoglycopeptides, showed dose-dependent bactericidal action. They prevent the synthesis of cell wall and interfere with the bacterial cell membrane’s permeability function. Therefore, the terminal acyl-d-alanyl-d is where the glycopeptide core binds. The alanine chain in the cell wall interacts with hydrophobic filling and hydrogen bonds resulting in a high affinity. This inhibits the cell wall precursors from polymerizing and crosslinking (Damodaran and Madhan, 2011). Three novel drugs from the lipoglycopeptide family have been approved and launched in the market. A lipoglycopeptide called dalbavancin was approved by FDA and European Medicine Agency (EMA) for treating the ABSSSI in 2014 and 2015, respectively (Van Bambeke, 2015). Dalbavancin is a semi-synthetic lipoglycopeptide with a long half-life ∼10 days and prolonged duration pf action ∼7 days against MRSA with a single dosage of 500 mg (Chen et al., 2007). Dalbavancin is specifically used to treat complex infections in outpatients (Juul et al., 2016). A multi-center study was conducted to treat ABSSSI in 2011–2012 to compare efficacy of dalbavancin with vancomycin. Compared to vancomycin, dalbavancin showed great outcomes with fewer side effects (Boucher et al., 2014).

Ritavancin, a second lipoglycopeptide, authorized by FDA and EMA in 2014 and 2015, which is also a prolonged action lipopeptide used to treat ABSSSI infections (Takahashi and Igarashi, 2018). This drug act by supressing the transglycosylase and transpeptidase enzymes. This antibiotic also exhibits bactericidal activity against broad range of gram-positive pathogens such as vancomycin-resistant enterococci (VRE), VISA, and VRSA due to its enhanced capability of penetration through plasma membrane (Van Bambeke, 2014). Telavancin is another lipoglycopeptdie which is quite effective against hospital-acquired pneumonia (HAP) caused by MRSA (Sandrock and Shorr, 2015). The drug is approved by FDA in 2013 for the treatment of MRSA infections including HAP and VAP following approval for SSSS treatment (Wenzler and Rodvold, 2015). When other antibiotics become ineffective, the EMA has restricted its usage for the treatment of nosocomial pneumonia caused by MRSA (Masterton et al., 2015). Although the lipoglycopeptides are approved for a narrow range of treatment of infections, in the future they will play important role in the treatment of osteomyelitis, bacteremia, and infective endocarditis (Brade et al., 2016).

Other antimicrobial drugs may include doxycycline, clindamycin, and trimethoprim/sulphamethoxazole which are also found effective irrespective of the severity of the disease (Liu et al., 2011).

### 8.2. Futuristic approaches to treat MRSA infections

As stated by World Health Organization (WHO), among the largest threat to public health, is the rise in antibiotic-resistant microorganisms. Approximately 700, 000 fatalities are caused by antibiotic resistance bacteria each year globally, and by 2050, that number might reach 10 million (Tagliabue and Rappuoli, 2018). A post-antibiotic era, in which ordinary diseases and mild infections might kill, is a very real prospect for the twenty first century, according to a study from WHO (Streicher, 2021). Thus, the development of novel antibiotic-free strategies is urgently required for the management and treatment of antibiotic-resistant bacterial infections.

#### 8.2.1. Herbal medicine

**FIGURE 5:**
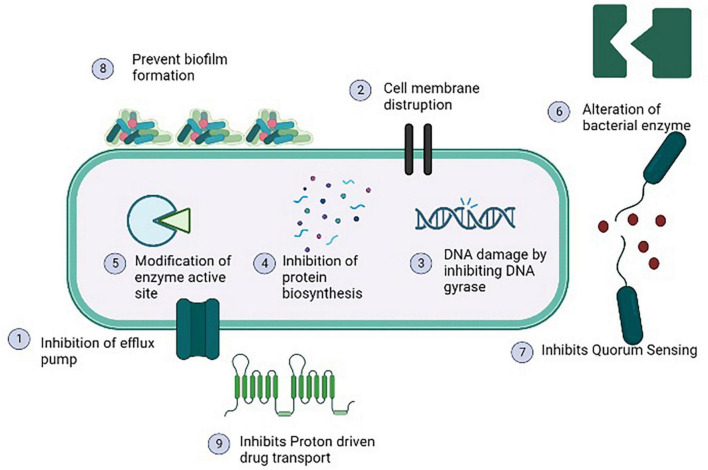
Antibacterial mechanisms of various phytochemicals against methicillin resistant strain of *Staphylococcus aureus* (MRSA).

The use of antibiotic stimulators in association with antibiotics is one of the best methods for reducing antibiotic resistance and extending the life of current antibiotics. The most effective combination against MRSA was found β-lactam antibiotic and potassium clavulanate (De Araújo et al., 2013). As listed in Table 3, many studies shown that phytochemicals alone or in combination with antibiotic have a great organic potential to exhibit antibacterial activity as well as act as modulators of antibiotic resistance. The majority of these curative effects are attributed to the active secondary metabolites produced by plants (Lakshmi et al., 2013). Phytochemicals act as antibacterial agent by inhibiting efflux pumps, modification of active sites, increasing the permeability of plasma membrane, and alteration of bacterial enzymes as depicted in Figure 5 (Coutinho et al., 2009). Plant extract along with gentamicin and kanamycin have synergistic effects for example ethanol extracted of *Turnera ulmifolia* leaves may enhance antibacterial efficacy of antibiotic against MRSA strains. As a possible active efflux regulator, grapefruit oil was found to be beneficial against MRSA (Abulrob et al., 2004). In an interesting study, the traditional Korean medicine known as Sami Hyanglyum-Hwan, *Aucklandiae radix*, *Coptidis rhizome*, *Rhei rhizome*, and *Arecae semen*) restored the anti-microbial activity of ciprofloxacin when evaluated against multiple MRSA strains (Choi et al., 2015).

**TABLE 3:** Antibacterial activity of different herbal plants against different strains of methicillin-resistant *Staphylococcus aureus* (MRSA).

Sr. No.	Plant name	Plant part	Extraction method	MRSA strains	ZOI or MIC (μg/ml)	References
1.	*P. nigrum* *H. cordata* *S. baicalensis* *C. sinensis*	Dried Fruit Stem + Leaf Roots Leaves	Sterile water	LA-MRSA	5,000 1,250 1,250 625	Guo et al., 2022
2.	*Z. album*	Arial parts	Ethanol extract	HA-MRSA	312.5–1,250	Sharaf et al., 2021
3.	*C. macrocarpa*	Leaves	Methanol, Ethanol, n-butanol	HA-MRSA	2.0–8.0 256–2,048	Attallah et al., 2021
4.	*C. longa*	Root	Hydroxypropyl methylcellulose	LA-MRSA	ZOI 10–18 mm	Sarwar et al., 2021
5.	*A. pavarii*	Leaf and Stem bark	Methanol	CA-MRSA	1.25–2.50	Buzgaia et al., 2020
6.	*O. lamiifolium* *R. officinalis* *C. roseus* *A. indica* *M. stenopetala*	Leaves	Diethyl ether, ethyl acetate, methanol, ethanol	HA-MRSA	Larger ZOI than antibiotics	Manilal et al., 2020
7.	*A. catechu* *G. mangostana* *I. balsamina* *U. gambir*	Wood Fruit Shell Leaf Leaf + stem	Ethanol	HA-MRSA	1.6–3.2 0.05–0.4 6.3 0.4–0.8	Okwu et al., 2019
8.	*C. sativa* *T. orientalis* *P. guajava*	Leaves	Ethanolic extract	HA-MRSA and CA-MRSA	Larger ZOI than antibiotics	Chakraborty et al., 2018
9.	*C*. *cyminum* *A*. *subulatum* *C*. *verum* *S*. *aromaticum*	Seeds Seeds Bark Buds	Hydro-distillation	MDR *S. aureus*	29.7 ± 1.7 9.4 ± 1.86 4.8 ± 0.96 5.4 ± 1.08	Naveed et al., 2013
10.	*S. exigua* *E. koreensis*	Root	Tetra-hydroxy Flavanones	HA-MRSA	3.13–6.25	Tsuchiya et al., 1996
11.	*G. glabra* *G. inflata* *G. uralensis*	Root	Flavonoids	HA-MRSA	3.13–12.50	Gupta et al., 2008
12.	*D. capitata* *E. rugulosa* *E. blanda* *G. strictipes* *P. multiflorum*	Apex Wall Apex Root Root	Ethanol extract Ethanol extract Ethanol extract Ethanol extract	HA-MRSA	1.25 1.43 1.32 1.34 1.34	Zuo et al., 2008
13.	*C. impressicostatum*	Sb	Water extract	HA-MRSA	19.50	Khan et al., 2009
14.	*P. betle*	Leaves	Ethanol extract	HA-MRSA	156–78	Valle et al., 2016
15.	*C. sinensis*	Leaves	Polyphenols	HA-MRSA	50–180	Choi et al., 2015
16.	*C. procera*	Leaves	Aqueous extract	HA-MRSA	12.5	Salem et al., 2014
17.	*E. globulus*	Leaves	Eucalyptus oil	HA-MRSA	4.0	Mulyaningsih et al., 2011
18.	*C. longa*	Root	Ethanol extract	HA-MRSA	217	Gunes et al., 2016

MIC, minimum inhibitory concentration.

#### 8.2.2. Synergistic effect of antibiotics with NSAIDs

Several studies have shown that NSAIDs exhibited antimicrobial properties, nevertheless, the exact mode of action is unclear. Except for mefenamic acid, it has been observed that diclofenac, aspirin, and ibuprofen have antibacterial effects at 5 mg/ml against certain gram-positive bacteria. Due to the presence of lipopolysaccharide in the cell wall of gram-negative bacteria, which is hydrophilic and inhibits most drugs metabolism, the only NSAID that is helpful against gram-negative bacteria is aspirin. The absence of lipopolysaccharide in cell wall of gram positive bacteria, makes it simple for antimicrobial drugs to enter the cells easily (Khalaf et al., 2015). In comparison to the typical therapeutic dosage in use for inflammatory, pain, or fever, NSAIDs have antimicrobial action at significantly lower concentrations (Ong et al., 2007). Opposite to diclofenac, aspirin and ibuprofen demonstrated bacteriostatic and bactericidal action against MRSA strains and may thus be used as antibiotic adjuvants to treat infections (Chan et al., 2017). The treatment of CA-MRSA infections involves the use of NSAIDs and antibiotics. Ineffective outcomes were seen when cefuroxime and chloramphenicol were taken alone to cure MRSA. Although aspirin and ibuprofen have bacteriostatic and bactericidal effects on MRSA strains, however their combination along with cefuroxime and chloramphenicol were examined. It was shown that the combination of ibuprofen/aspirin, chloramphenicol, and cefuroxime have increased antibacterial showing either synergistic or additive effect (Yin et al., 2014). MDR bacterial infections can be treated with NSAID and antibiotic combination (Chan et al., 2017). Another study conducted by Aqib et al. (2021) investigated the effect of antibiotics alone, in combination with NSAIDs, nanoparticles and plant extracts against MDR strains of *S. aureus* including MRSA. It was noted antibiotic in combination with NSAIDs such as meloxicam, diclofenac, aspirin and ibuprofen increases the ZOIs in *in vitro* studies against MRSA and MDR strains of *S. aureus*. The study concluded synergistic correlation between NSAIDs, antibiotics, nanoparticles and plant extracts by calculating fractional inhibitory concentration indices (FICIs).

#### 8.2.3. Nanoparticles as therapeutic agents

Due to the special characteristics of metal nanoparticles (NPs), they are more ubiquitous and inexpensive manufacturing material that are getting much applications in today’s world. In 2016, it was noted that the nanometals market based on metal oxides reached USD 4.2 billion. A more rise in NPs manufacturing demand is found to be anticipated by 2025, which is due to increased use of metal based nanomaterials in biomedical research (Gudkov et al., 2022). Research studies going on to explore the potential of nanoparticles as biosensors (Singh et al., 2019), detection and treatment of oncological disorders (Vimala et al., 2019), and drug delivery have received a lot of interest (Spirescu et al., 2021). It is very interesting to employ metal oxide nanoparticle-based on nanomaterials to treat antibiotic resistant bacterial infections. Now a day, most commonly used metal oxide nanomaterials are silver nitrate, zinc oxide, platinum, aluminum oxide, titanium dioxide, gold, magnesium oxide, iron oxide and sodium alginate (Akhtar et al., 2019; Spirescu et al., 2021; Gudkov et al., 2022; Mendes et al., 2022).

**FIGURE 6:**
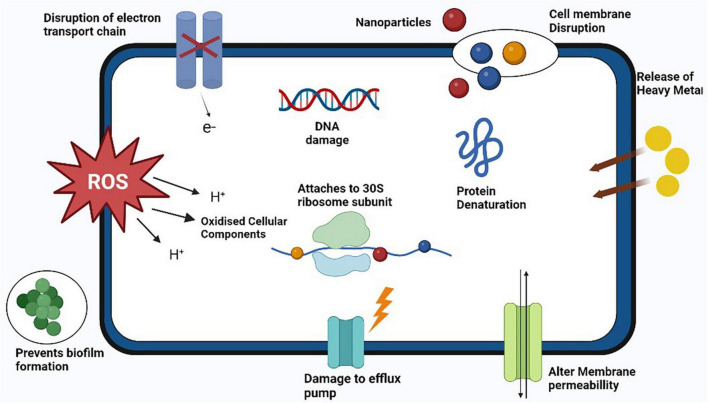
Antibacterial mechanisms of action of various nanoparticles against methicillin resistant strain of *Staphylococcus aureus* (MRSA).

According to the statement from WHO, antibiotic-resistant microorganisms are among the most remarkable barriers to public health and progress. The new paradigm to treat the diseases caused by resistant bacterial strains including MRSA is the use of metal oxide nanomaterials [95]. The NPs act on bacterial cell through various type specific mechanisms such as production of reactive oxygen species (ROS) to induce stress on cell, release of heavy metal ions, alter membrane permeability, DNA damage, protein damage, disrupt the function of efflux pump, act of cell wall and plasma membrane to cause damage and release cellular components outside of cell (Fernando et al., 2018) as illustrated in Figure 6. (Shameli et al., 2010) conducted study to evaluate the antibacterial activity of green silver nitrate nanoparticles against MRSA isolates and found that green silver nitrate nanoparticles exhibited the strong antibacterial activity by inhibiting the growth of MRSA in *in vitro* model. Another study was conducted by Lodhi et al. (2021) who evaluated the antibacterial activity of zinc oxide NPs alone and in combination with antibiotic and found increased antibacterial activity when NPs were used in combination with antibiotic. Another study conducted by Wichai et al. (2019) in Thailand who use a complex combination of nanomaterials with other materials (bacterial cellulose + sodium alginate NPs + chitosan + copper sulfate) and found that NPs exhibited a strong antibacterial activity against MRSA strains. A number of studies has been conducted by various researchers from different countries to check the antibacterial activity of various forms of nanoparticles against different strains of *S. aureus* including MRSA are listed in Table 4.

**TABLE 4:** *In vitro* studies on antibacterial activity of nanoparticles against methicillin resistant strain of *Staphylococcus aureus* (MRSA).

Sr. No.	Composition of NPs	MRSA strain	ZOI or MIC	Country	References
1.	Green silver nitrate	HA-MRSA	8.4–8.8 mm	Malaysia	Shameli et al., 2010
2.	Polyacrylonitrile copper oxide	CA-MRSA	7.0 ± 0.05 mm	Taiwan	Wang and Clapper, 2022
3.	Green platinum NPs	MRSA	1.0 μg/ml	Egypt	Eltaweil et al., 2022
4.	Chitosan-gold NPs-Plant extract	MRSA	15.6 μg/ml	Egypt	Hussein et al., 2021
5.	Zinc oxide Zinc oxide + antibiotic	LA-MRSA	125 μg/ml 10.42 μg/ml	Pakistan	Lodhi et al., 2021
6.	Nickle oxide	HA-MRSA	265 μg/mL	Egypt	Rheima et al., 2021
7.	Iron Oxide (IO) NPs IO NPs + Vancomycin IO NPs + Ceftriaxone IO NPs + Gentamicin	HA-MRSA	12 ± 0.21 mm 25 ± 0.3 mm 37 ± 0.21 mm 34 ± 0.2 mm	Malaysia	Majeed et al., 2021
8.	Biosynthesized silver nitrate NP + vancomycin	MRSA	0.39 ± 0.16 mm	Egypt	Awad et al., 2021
9.	Titanium dioxide + erythromycin	HA-MRSA	2–16 mg/L	Pakistan	Ullah et al., 2020
10.	Endolysins + sodium alginate + chitosan NPs	MRSA	22.5 ± 3.1 mm	India	Kaur et al., 2020
11.	Bacterial cellulose + sodium alginate NPs + chitosan + copper sulfate	MRSA	5.0 mm at 0.9 conc.	Thailand	Wichai et al., 2019
12.	Zinc oxide	HA-MRSA	312.5–1,250 μg/ml	India	Umamageswari et al., 2018
13.	Silica silver nitrate	HA-MRSA	2.5–5.0 μg/ml	Taiwan	Chien et al., 2018
14.	Magnesium oxide	MRSA	1.0 μg/ml	USA	Nguyen et al., 2018
15.	Aluminum oxide	MDR-MRSA	1,700–3,400 μg/ml	India	Ansari et al., 2013

NPs, nanoparticles; MIC, minimum inhibitory concentration; ZOI, zone of inhibitions.

#### 8.2.4. Bacteriophages as an alternate to antibiotics

**FIGURE 7:**
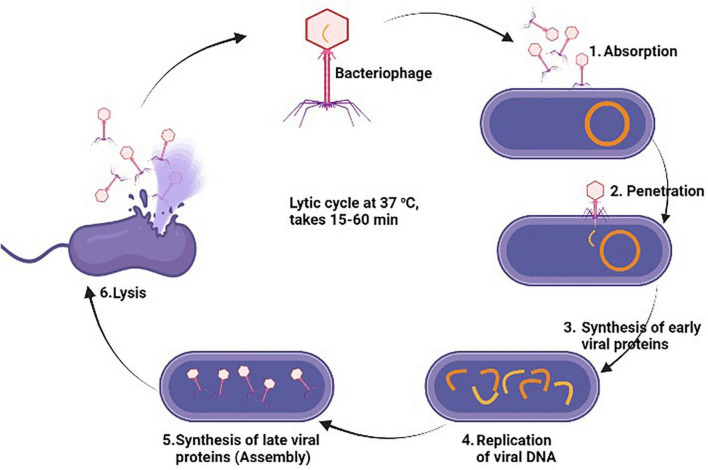
General antibacterial mechanism of bacteriophages against methicillin resistant strain of *Staphylococcus aureus* (MRSA).

Bacteriophage therapy also known as phage therapy is a type of therapy that uses viruses to kill bacterial pathogens. Due to increasing resistance to antibiotics, phage therapy is considered as cost effective, highly specific and efficient in their mechanism of action against multiple MDR bacteria. Bacteriophages only cause damage to bacteria cells without causing any damage to human and animal cells (Tkhilaishvili et al., 2020). Phage therapy has been found as an effective treatment against multiple bacterial pathogens such as *S. aureus*, *E. coli*, *P. aerogenosa*, *A. baumannii*, *S. pyogenes*, *S. suis*, and *B. cereus* (Yang et al., 2017). This therapy is mostly used in Georgia and Russia for the treatment of those bacterial infections which don’t response to antibiotics. A study was conducted by Nandhini et al. (2022) who evaluated the lytic activity of kayvirus phages against MDR *S. aurus*. (Lubowska et al., 2019) also study the genomic, morphology, and lytic properties of three phages which are name as vB_SauM-A, vB_SauM-C, and vB_SauM-D and noted rapid adsorption with bacterial cell, short latent period, and increase lytic activity. Further genome study showed higher G + C content in these phages similar to phage K (Nandhini et al., 2022). As, bacteriophage act so specifically consist of following steps to destroy bacterial cell; (1) Adsorption which is attachment of virus with specific bacteria cell, (2) Penetration of phage DNA or RNA into the bacteria cell, (3) after penetration, phage uses host cell machinery to synthesis early viral proteins, (4) Replication of virus through using the early viral proteins, (5) Synthesis of late viral proteins to assemble again in new complete phage particles, (6) Lastly, new phage particles cause lysis of the bacterial cell and causes its death through release of cellular contents outside the cell and new phage particle starts infecting the bacterial cells (Sausset et al., 2020) as illustrated in Figure 7. Staphylococcal bacteriophage (Sb) and PYO bacteriophage are prepared by Eliava Institute, Georgia and available commercially as phage preparations for usage as replacement to antibiotic by humans (Fish et al., 2018). Bacteriophage Sb is a mono phage product and is effective against only one specific bacteria while PYO phage covers multiple bacterial species such as *E. coli*, *S. aureus*, *Streptococcus* spp., *P. aeruginosa*, and *Proteus* spp (Villarroel et al., 2017). The number of isolated phages against MRSA has significantly increased over the past 15 years, and several investigations have found that phages have effective and all-encompassing antibacterial effects as listed in Table 5.

**TABLE 5:** *In vitro* studies on antibacterial activity of bacteriophages against methicillin resistant strain of *Staphylococcus aureus* (MRSA).

Sr. No.	Bacteriophage	Phage isolation source	Phage efficacy assessment assay	Host range	Antibacterial activity method and result	Country	References
	Phage Rih21	Hospital Wastewater	Double layer agar method	MRSA	showed lytic activity	Iraq	Ghayyib et al., 2022
	Phage cocktail APTC-C-SA01	Nasal swabs, soil and sheep feces samples	Plaque assay	Biofilm MRSA and MSSA	> 98% lytic activity	Australia	Liu et al., 2022
	Phage Henu2 + Antibiotics	Sewage sample	Time kill assay	MRSA, MSSA	Growth decreased faster	China	Li et al., 2021
	Phage Sb1 + antibiotics	Commercial purchased	Plague assay and time kill assay	MRSA	Decrease in CFUs after treatment	USA	Kebriaei et al., 2020
	Recombinant chimeric bacteriophage endolysin HY-133	Hypharm GmbH	Broth micro dilution method	MRSA, MSSA, mupirocin-resistant strains	MIC = 0.12–0.5 mg/L, Time kill curve assay showed Bactericidal effect	Germany	Knaack et al., 2019
	Staphylococcal Sb and PYO bacteriophage	Eliava Biopreparations	Microcalori-metry Assay and CFU counting	MRSA	Rapidly inhibited growth of MRSA in both methods	Germany	Tkhilaishvili et al., 2020
	Sb-1 phage	Georgia Eliava Institute	Spot assay	MRSA. MSSA, biofilm	22/28 MRSA sensitive, 16/29 MSSA sensitive, eradicated biofilm	Germany	Tkhilaishvili et al., 2018
	Chimeolysin F (ClyF)		96-well plate method	*S. sureus* multiple strains, MRSA, *S. pyogenes, S. suis, B cereus*	Lytic activity against all *S. aureus* strains and no against other strains	China	Yang et al., 2017
	pq/27 and pq/48	Sewage water	Spot assay	MRSA	Agar method +	Pakistan	Rasool et al., 2016
	Lysostaphin + L. monocytogenes bacteriophage endolysin-ply511	N/A	Peptidoglycan hydrolytic activity	MRSA	Agar method High bactericidal activity	Australia	Turner et al., 2007

#### 8.2.5. Probiotics as therapeutic agents

**FIGURE 8:**
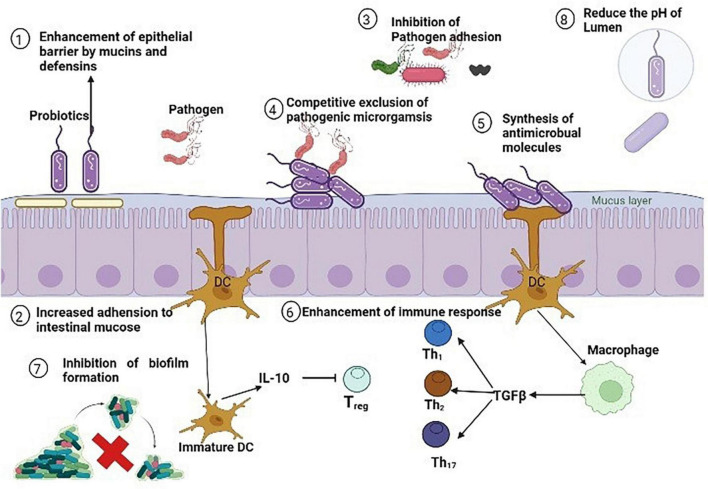
Antibacterial mechanisms of various probiotics against methicillin resistant strain of *Staphylococcus aureus* (MRSA).

Utilizing probiotics is a prospective antibiotic substitute. Probiotics are live microorganism known to aid during infection or after antibiotic therapy which upsets the normal gut microbiota. Additionally, they could help to relieve certain other conditions such as irritable bowel syndrome (IBS) symptoms and antibiotic associated diarrhea (McFarland et al., 2021). Probiotic can improve the health of many other body tissues by replenishing their respective microbiota and releasing anti-pathogenic chemicals. So, the word probiotic is not only restricted to gut microbiota but they also provide various other health benefits such as enhances the host immunity by upregulating the immune cells, depression and anxiety disorders, tumor suppression, therapeutic role in COVID-19, overweight and obese patients (Herman, 2019; Shi et al., 2019; Shin et al., 2019; Ceccarelli et al., 2020; Daniali et al., 2020). Firstly, specific strains of Lactobacilli were used and evaluated for antimicrobial properties. Nowadays, many non-pathogenic species of bacterial as well as fungal genera are used as potential source of probiotic such as Streptococcus, Bifidobacterium, Bacillus, Escherichia, Enterococcus, and Saccharomyces from fungi. Various strains of Lactobacillus (*L. rhamnosus, L. plantarum, L. animalis*, *L. reuteri*, *L. lactis, L. gasseri, L. curvattus, and L. lacis*), Bacillus (*B. subtilis*, *B. amyloliquefaciens*), Bifidobacterium (*B. lactis, B. bifidum, B. breve, B. dentium, B. longum, B. infantis, B. catenulatum, B. pseudo catenulatum*), and Saccharomyces (*S. cerevisiae*) are all summarized in Table 6. All of the probiotic act by adopting one or more than one mechanism from the following; enhancement of epithelial barrier, increased adhesion to intestinal mucosa, synthesis of antimicrobial molecules, inhibition of pathogen adhesion to intestinal cells, competitive exclusion of pathogenic microorganisms, reducing the pH of gut lumen, and enhancement of immune response (Plaza-Diaz et al., 2019) as illustrated in Figure 8.

**TABLE 6:** *In vitro* studies on antibacterial activity of probiotics against methicillin resistant strain of *Staphylococcus aureus* (MRSA).

Sr. No.	Probiotic name/Microbial strain	Evaluation assay	Source of pathogenic organism	Test organisms	Antibacterial effect	Country	References
	Probiotic cellulose	Agar diffusion assay	Colección Española de Cultivos and Urine	*S. aureus*, *P. aeruginosa*, MRSA	Inhibited growth of all	Spain	Sabio et al., 2021
	Chitosan encapsulated strains of *L. lactis* and *L. curvattus*	Agar diffusion assay	Pus, urine, blood	*S. pyogenes, E. coli, K. pneumoniae, S. epidermidis, S. aureus, P. aeruginosa, and S. marcescens*	ZOI shown antibacterial activity against all pathogenic strains	Pakistan	Nasreen et al., 2022
	*B. subtilis* KATMIRA1933, *B. amyloliquefaciens* B-1895	Agar well diffusion assay	Wound infection	MRSA and MSSA	Growth inhibition seen as ZOI	Iraq	Algburi et al., 2021
	Bifidobacterium strains (*B. lactis, B. bifidum, B. breve, B. dentium, B. longum, B. infantis, B. catenulatum, B. pseudo catenulatum*)	Agar well diffusion and dilution assays, MIC micro-dilution assay	Sahmyook medical center	MDR *S. aureus* ATCC 25923, *P*. *aeruginosa* ATCC 27853, *E. faecalis* ATCC 29212	ZOI were observed, lower MIC than antibiotics	Korea	Choi and Shin, 2021
	*C. accolens*	Agar well diffusion assay	Chronic rhinosinusitis patients	*S. aureus* ATCC 25923, MRSA, MSSA	ZOI of inhibitions indicated inhibitory growth	Australia	Menberu et al., 2021
	*B. subtilis*	Agar radial and spot assays	Animal Health Lab at UOG	Enterotoxic *E. coli* (ETEC), *S. typhimurium*, MRSA	ZOI were observed against all bacterial strains	Canada	Sudan et al., 2021
	*L. plantarum* CRL 759	Agar slab method, Agar diffusion assay, Optical density method	Human diabetic foot	*P. aeruginosa*, MRSA	Inhibited growth	Argentina	Layus et al., 2020
	Lactic acid bacteria	Agar well diffusion assay	Dairy animals	MRSA	ZOI were observed 16–29 mm	India	Essayas et al., 2020
	*L. gasseri* YIT 12321	Radial diffusion assay	Respiratory patient	MRSA	ZOI showed growth inhibition	Japan	Ishikawa et al., 2020
	*L. animalis* 30a-2, *L. reuteri* 4-12E, *L. lactis* 5-12H, *W. cibaria* C34	Agar well diffusion assay	FIRDI, Hsinchu, Taiwan; Chang-Hua Hospital in Taiwan	MRSA, ESBL *E. coli*, *P. aeruginosa*, *B cereus* ATCC 1178, *L. monocytogenes* ATCC 19111, *Y. enterocolitica* BCRC 12986, *S. choleraesuis* ATCC 13312, *S. enteritidis* ATCC 13076, *S. typhimurium* ATCC 13311, *S. fexneri* ATCC 29903, *S. sonnei* ATCC 25931	Represses the growth of all strains	Taiwan	Lin et al., 2020

## 9. Futuristic approach to prevent MRSA infections

Currently, many MRSA prevention and control interventions are carried out such as judicial use of antimicrobials, hand hygiene, controlling the interaction with *S. aureus* natural reservoirs, preventing transmission from infected patient, decolonization, isolation, disinfection of hospital environment, active surveillance, and many other (Lee et al., 2018). Now there is a need to develop most effective way to control MRSA at animal and human cadre i.e., vaccine production. The problem of antibiotic resistance including MRSA pointing to a notable concern and is under study by Center for Disease Control and Prevention and World Health Organization (Klevens et al., 2007). The development of novel ways to combat antibiotic resistance and production of vaccine very important in this era. MRSA is not well-known to be resistant to any known antibodies (Anderson et al., 2012). However, attempts to develop a potent vaccine against MRSA are under trial by various companies (Adhikari et al., 2012). To develop a potent vaccine against multiple MRSA strains, there is need to use multiple antigens to develop effective immunity against different strains (Aqib et al., 2018a). Additionally, an appropriate adjuvant needs to be added to the vaccine to improve the vaccine function as well as delivery into the host (Adamczyk-Poplawska et al., 2011). To create an effective multi-epitope component immunization against staphylococcal infections, three antigenic determinants are known to be much important which are clustered factor A (ClfA), alpha-enolase (Eno1), and iron regulated surface determinant protein B (IsdB). Eno1 is a polypeptide present in cytoplasm of cell and found in all *S. aureus* strains and has a very well-preserved lineage. Additionally, this protein aids in the mechanism of adhesion and contributes to the spread of infection, so this protein is a potential candidate for vaccine development against multiple *S. aureus* strains (Ghasemi et al., 2016). ClfA, another cell surface protein, aids in the pathogen’s adherence to the host. Previous investigations have shown that ClfA plays a key role in the development of staphylococcal illnesses (Garcia-Lara and Foster, 2009). Thus, to initiate a strong, active, and independent immune response to *S. aureus*, it is important to include this surface component as a vaccine agent (Brouillette et al., 2002). Another surface protein that aids in attachment to the cell membrane is IsdB, the third epitope marker (Zapotoczna et al., 2013).

A vaccine is an agent that prevent the infection before its onset and also disrupt the colonization of infection causing organism with host cell and thereby act as long lasting infection prevention agent and extensively reduces the use of antibiotics (Pozzi et al., 2017). A monoclonal antibody is developed by Medimmune, USA based company against α-haemolysin factors of *S. aureus* which may provide protective immunity administered alone or in combination with antibiotics. Another two monovalent trial based vaccines were prepared by USA based company and tested but failed to generate protective levels at the later stages of development. Then two more vaccines StaphVax and V710 were formulated by Nabi Pharmaceuticals, USA containing capsular polysaccharides CP5 plus CP8 and IsdB respectively as an antigenic component. Both of the vaccines provided immunity during animal model but failed in control phase III trials (Shinefield et al., 2002). The failure may be due to certain strains of *S. aureus* such as USA 300 does not contain CPs in their structure, lack of adjuvant in vaccine preparation, *S. aureus* immune evasion virulence factors such as IgG binding protein A have ability to compromise the function of antibodies and make them unable to provide effective immunity (Fowler and Proctor, 2014). However, the trials are underway to make a polyvalent vaccine containing number of antigens such as ClfA, CP5, CP8, secreted toxins (extracellular protein A and B, α-toxin, ESAT-6, *luk*S-PV), MntC, and Fhud2 (Pozzi et al., 2017). Another monoclonal vaccine containing WTA targeted antibodies plus rifampicin class antibiotic were tested and found protective effects in preclinical trials (Lehar et al., 2015). Moreover, further research is going on and hope soon a better vaccine will be able in market to treat multiple *S. aureus* strains including MRSA.

## 10. Conclusion

Methicillin resistant strain of *S. aureus* (MRSA) is found to be versatile and unpredictable pathogen with diversity of lineages common between humans and animals indicated its transmission between human and animals. The lineages found common between human and animal were CC398, CC9, CC130, CC97, CC398. Except this, few HA-MRSA and CA-MRSA lineages were identified in animals and LA-MRSA lineages were identified similar to HA-MRSA and CA-MRSA. Therefore, increasing prevalence and genetic adaptation of this pathogen at animal and human cadre exposing it a major threat for public health. This pathogen holds a diversity of host species ranging from humans to food and companion animals and many more. This pathogen has the ability to resist many antibiotics and to escape immune mechanisms through various virulence factors that unable this pathogen to cause mild to life threatening infections. Due to the increasing antibiotics resistance, this article highlighted the importance of alternative ways such as phytochemicals, bacteriophages, nanoparticles, and probiotics alone and in combination to use them as replacement of antibiotic. There is need to work more on these approaches to combat antibiotic resistance and making them available at commercial scale for public. One more approach is to work on successful vaccine production and immunization against MRSA.

## Author contributions

MS and AA wrote the whole manuscript. WP structured and funded the project. IM and MK review the manuscript. ZB, AM, MF, and MM drew the illustrations. C-NZ, MK, FS, and NM added the references through endnote. All authors contributed to the article and approved the submitted version.
